# Revisiting Defect‐Engineered M(III)‐Doped ZnO Photocatalysts for Emerging Pollutant Photodegradation and Mineralization: A Mini‐Review

**DOI:** 10.1002/cphc.70423

**Published:** 2026-05-19

**Authors:** Abderrahmane Toutlitni, Jamal Khmiyas, Sara Fatine, Jean‐Michel Nunzi, Abdelaziz Laghzizil

**Affiliations:** ^1^ Laboratory of Applied Chemistry of Materials Faculty of Sciences Mohammed V University in Rabat Rabat Morocco; ^2^ Department of Physics, Engineering Physics and Astronomy Department of Chemistry Queen's University Kingston Canada

**Keywords:** metal doping strategies, oxygen vacancies, photocatalytic degradation, water purification, ZnO‐based photocatalysts

## Abstract

Zinc oxide (ZnO) is a widely studied semiconductor for photocatalytic applications due to its suitable bandgap, chemical stability, and high electron mobility. Its photocatalytic efficiency is often limited by rapid electron–hole recombination. Metal doping has emerged as an effective strategy to overcome this limitation as it introduces lattice distortions, modifies electronic properties, and creates oxygen vacancies. Trivalent metal dopants substitute Zn^2+^ in ZnO lattice, inducing oxygen vacancies that act as intrinsic defects, trap charge carriers, extend their lifetime, and enhance charge separation efficiency. Moreover, the presence of V_O_ facilitates the generation of reactive oxygen species (ROS), such as superoxide radicals (^•^O_2_^−^) and hydroxyl radicals (^•^OH), under UV or visible light irradiation, which radicals are responsible for the oxidative degradation of organic pollutants. The synergy between metal dopants and oxygen vacancies not only tunes the optical and electronic properties of ZnO but also significantly improves its photocatalytic performance. This makes doped ZnO an attractive candidate for water treatment applications, including the degradation of pharmaceuticals, dyes, and other persistent organic contaminants. Understanding the interplay between dopant type, vacancy concentration, and ROS generation is crucial for designing highly efficient, stable, and recyclable photocatalysts for sustainable water purification.

## Graphical abstract

Water pollution has become a major environmental concern due to industrial, agricultural, and urban activities that release various pollutants into freshwater systems. ZnO‐based photocatalysis is an effective strategy for organic pollutant degradation, but its performance is limited by rapid electron–hole recombination and weak adsorption capacity. Metal doping has emerged as an efficient approach to overcome these limitations.

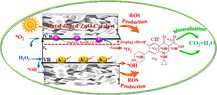

## 1 Introduction

Water pollution caused by organic contaminants has become a major environmental problem worldwide, as these compounds, including pesticides, dyes, and pharmaceuticals, are generally persistent and poorly removed by conventional treatment technologies [1, 2, 3]. Their presence in aquatic environments has toxic effects on aquatic organisms and can have significant consequences for human health. Among these emerging pollutants, dyes and antibiotics are of particular concern. Among water treatment methods, heterogeneous photocatalysis has proven to be an effective solution for the complete degradation and mineralization of these micropollutants [4, 5].

Various representative semiconductor photocatalysts, including graphitic carbon nitride TiO_2_, WO_3_, Fe_2_O_3_, CeO_2_, Bi_2_O_3_, g‐C_3_N_4_, BiVO_4_, SnO_2_, ZnO, CdS, and ZnS have been widely studied for pollutant degradation under UV and/or visible‐light irradiation due to their diverse band structures, optical properties, and redox behaviors [6, 7]. However, each material presents specific limitations, such as rapid charge recombination, limited light absorption, or insufficient charge transfer efficiency. Semiconductors such as TiO_2_ and its derivatives are widely used because of their chemical stability, efficiency under UV light, and low toxicity [8, 9]. In addition, ZnO has been widely investigated as a promising alternative to the conventional benchmark TiO_2_ photocatalyst due to its attractive physicochemical and optical properties and remains the most extensively studied photocatalyst in environmental remediation. Compared to TiO_2_ photocatalyst, ZnO offers several advantages, including lower cost, simple and versatile synthesis methods, and high natural abundance. In addition, ZnO exhibits a wide direct bandgap (≈3.20 eV), high exciton binding energy (≈60 meV), and strong redox potential, making it highly efficient for the generation of reactive oxygen species (ROS) under UV irradiation [10, 11]. Importantly, ZnO maintains a single thermodynamically stable phase even under relatively high temperatures, which is advantageous for catalyst recycling and repeated use without significant structural transformation. Its flexible morphology and surface chemistry further enhance its suitability for environmental remediation applications.

Despite these advantages, ZnO also suffers from rapid recombination of photoexcited electrons and holes, as well as limited absorption of visible light, which motivates extensive research on its modification strategies, particularly metal ion doping, to improve its photocatalytic performance. Metal doping is among the solutions to overcome these limitations and has been the subject of numerous studies [12, 13]. The introduction of metal ions into the ZnO crystal lattice significantly alters its electronic structure and promotes the formation of oxygen vacancies, which play a central role in enhancing photocatalytic activity. Among the various metal dopant ions, M^3+^ ions exert a more pronounced influence on the crystal structure and electronic configuration of the ZnO lattice compared to M^+^ and M^2+^ ions, as their higher charge state induces stronger lattice distortion and defect formation [14]. This enhanced structural perturbation promotes a higher density of oxygen vacancies and defect levels, which act as effective trapping sites for photogenerated charge carriers, thereby significantly suppressing electron–hole recombination. In addition, M^3+^ doping more effectively modulates the band structure of ZnO, leading to improved charge separation and, in some cases, a slight narrowing of the bandgap, which facilitates better utilization of light irradiation. Consequently, ZnO doped with trivalent metal ions generally exhibits superior photocatalytic performance in comparison with monovalent and divalent doped systems, owing to its more efficient generation of ROS and enhanced redox activity. Their incorporation induces a spectral shift toward the visible, associated with the appearance of intermediate energy states within the bandgap, which leads to a reduction in the effective bandgap energy. This allows the doped material to absorb a larger portion of the solar or artificial visible spectrum, thus significantly improving its performance under realistic irradiation. M^3+^ doping also improves the separation of photoexcited charges, oxygen vacancies, and energy levels and acts as a trap for electrons or holes, slowing down their recombination. The extension of charge carrier lifetime plays a crucial role in improving the photocatalytic efficiency of ZnO, as it significantly reduces the rate of electron–hole recombination and thereby increases the probability that photogenerated electrons and holes participate in surface redox reactions. As a result, more charge carriers reach the catalyst surface and are effectively involved in the formation of ROS. This enhanced charge separation leads to a higher production of highly reactive species, particularly hydroxyl radicals (^•^OH) and superoxide radicals (^•^O_2_^−^), which are the primary oxidizing agents responsible for the degradation of organic pollutants. These reactive species attack pollutant molecules through a series of oxidative pathways, ultimately leading to their fragmentation, decolorization, and complete mineralization into environmentally benign products such as CO_2_, H_2_O, and inorganic ions.

Depending on the nature and concentration of the dopant, the bandgap can be slightly reduced or adjusted, thus optimizing photon absorption for different wavelengths and adapting the photocatalyst to specific lighting conditions. M^3+^ doping therefore not only introduces structural defects but also tailors the absorption of ZnO to visible light, thereby improving overall photocatalytic efficiency and pollutant mineralization. Several recent reviews discuss ZnO‐derived materials and their numerous applications, including photonics, luminescence, catalysis and photocatalysis, water treatment, sensors, electronic devices, and antibacterial coatings [15]. However, these studies do not always clearly specify the type of modification applied to the ZnO, which can be metal doping, solid solutions, or composites. This lack of clarity sometimes complicates the comparison of performance and the identification of the mechanisms responsible for the improvement of properties, such as increased oxygen vacancies, enhanced visible light absorption, or reduced electron–hole recombination [16].

This review aims to provide a comprehensive analysis of recent research on metal‐doped ZnO, particularly trivalent metals, focusing on several key aspects. It first examines the role of vacancies and doping‐induced energy levels, which modify the electronic structure of ZnO and enhance the separation of photoexcited charges, thereby promoting the generation of reactive radicals responsible for photocatalysis. Next, it details the degradation and mineralization of certain organic pollutants such as dyes and antibiotics, highlighting how M^3+^‐doped ZnO can not only fragment these molecules but also efficiently convert them into harmless products. Particular attention is also paid to comparing the performance of different dopants, depending on the ion type, concentration, and synthesis methods, in order to identify the most effective strategies for maximizing photocatalytic activity. Finally, this review addresses the prospects and challenges related to the practical applications of these photocatalysts, including their stability, recyclability, and adaptation to real irradiation conditions, thus providing a framework to guide future research and the development of more efficient and sustainable water treatment systems.

## 2 Fundamental Properties of ZnO

### 2.1 Structure

**FIGURE 1:**
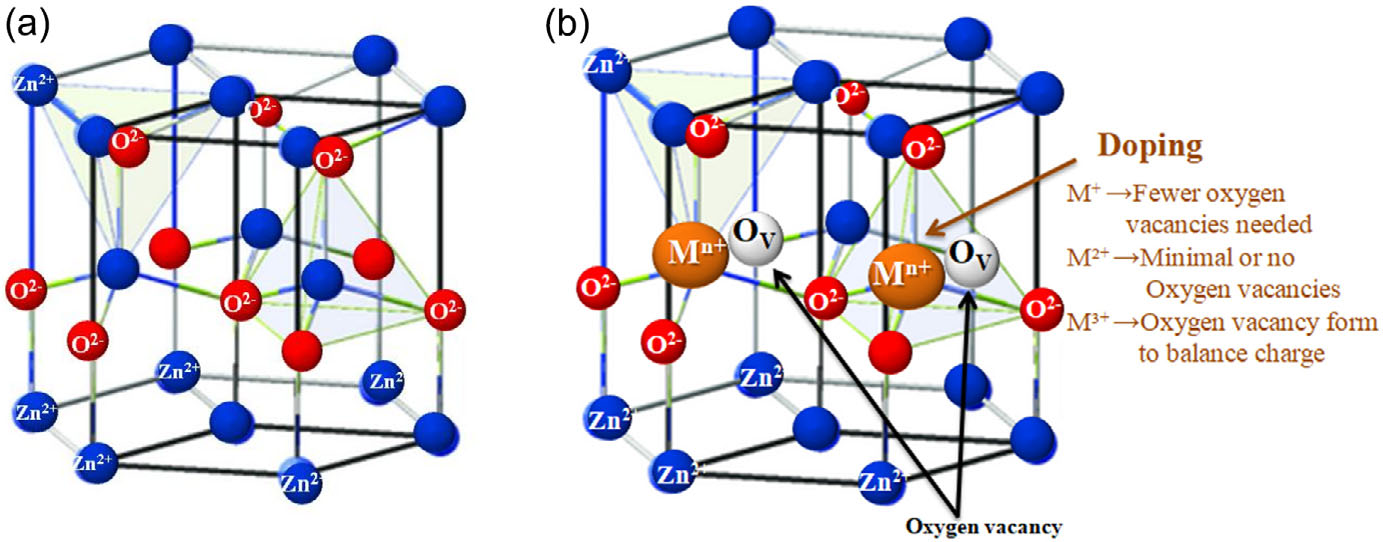
Wurtzite lattice of ZnO (a) and metal‐doped ZnO (b) showing the substitution of Zn^2+^ by M^+^, M^2+^, and M^3+^ as well as the compensating formation of an oxygen vacancy.

ZnO is an II–VI semiconductor widely used in photocatalytic, environmental, and electronic applications [15, 16]. It primarily crystallizes in the hexagonal wurtzite (P6_3_mc) structure, although cubic zinc blende (F‐43m) forms can occur depending on the synthesis conditions. In the wurtzite structure, each zinc atom is tetrahedrally coordinated to four oxygen atoms, and each oxygen atom is bonded to four zinc atoms, forming a stable three‐dimensional lattice with typical lattice parameters *a* ≈ 3.25 Å and *c* ≈ 5.2 Å. The structure is polarized along the *c*‐axis, generating an internal electric field that partially promotes electron–hole pair separation under irradiation, an important mechanism for photocatalysis. This configuration confers good mechanical and chemical stability to ZnO, as well as a surface rich in natural hydroxyl (–OH) sites that facilitate interactions with polluting molecules [10, 11]. Figure 1 illustrates the wurtzite crystal structure of pure ZnO (a) and metal‐doped ZnO (b). In the doped lattice, the partial substitution of Zn^2+^ ions by metallic ions (M^+^, M^2+^, and M^3+^) induces a charge imbalance, which is compensated by the formation of oxygen vacancies that critically influence the electronic structure and photocatalytic performance of the material.

A comparative analysis of ZnO doped with mono‐, di‐, and trivalent metal ions reveals distinctly different influences on structural, textural, and electronic characteristics, as well as on the potential for solid solution formation. Monovalent dopants (M^+^) partially replace Zn^2+^ ions, with charge compensation achieved through the generation of oxygen vacancies. This results in slight distortions of the wurtzite lattice while preserving phase purity. In contrast, divalent ions (M^2+^), due to their identical charge to that of Zn^2+^, incorporate more readily into the crystal structure, favoring the formation of homogeneous solid solutions with only slight variations in lattice parameters.

Trivalent dopants (M^3+^) induce greater perturbations of the crystal lattice, characterized by increased microstrains, reduced crystallite size, and a higher concentration of structural defects, particularly oxygen vacancies. The stability of these solid solutions is strongly influenced by the ionic radius and the solubility limit of the dopant in ZnO. From a textural perspective, the incorporation of M^+^ and M^3+^ tends to inhibit crystal growth, leading to finer particles and increased specific surface area, while doping with M^2+^ largely preserves a regular morphology and moderate porosity. From an electronic perspective, monovalent and especially trivalent dopants introduce defect‐related states into the ZnO bandgap, extending optical absorption into the visible range and modulating charge carrier density. Divalent ions, on the other hand, primarily play a structural role, with a limited electronic impact. Ultimately, the dopant valence and ionic radius govern defect formation, microstructural development, and solid solution stability, which are key factors in optimizing photocatalytic performance. As summarized in Table 1, these effects encompass structural and textural modifications, electronic alterations, and charge compensation mechanisms described by the Kröger–Vink notation. Although pure ZnO has a moderate specific surface area, its favorable electronic structure, polar network, and surface hydroxyl groups promote adsorption and photocatalytic reactions; however, its activity is highly sensitive to particle size, morphology, and defect concentration. Therefore, metal ion doping combined with oxygen vacancy engineering, particularly by the incorporation of M^3+^, appears to be an effective strategy for improving visible light absorption, charge separation, and reactive species generation, thus greatly improving ZnO‐based photocatalysts for water treatment applications.

**TABLE 1:** Comparative effects of mono‐, di‐, and trivalent metal ion doping on ZnO, showing solid solution type, charge compensation (Kröger–Vink), and the resulting structural, textural, and electronic modifications [17, 18].

Dopant	Solid solution	Charge compensation (Kröger–Vink)	Structural	Textural	Electronic
M^+^ a	Zn_1−*x*_M^+^_*x*_O_1−*x*/2_ wurtzite lattice distortion	Formation of oxygen vacancies M_Zn_^+^ + V_O_••	Slight distortion of the wurtzite lattice	Inhibition of crystal growth, smaller grain size	Introduction of intermediate states in the bandgap, redshift of light absorption toward visible
M^2+^ b	Zn_1−*x*_M^2+^_*x*_O homogeneous	No charge compensation required M_Zn_^+^	Minor changes in lattice parameters. Crystallinity preserved	Regular morphology, moderate porosity	Limited electronic effect, few intermediate states created
M3+^c^	Zn_1−*x*_M^3+^_*x*_O_1+*x*/2_ defect‐rich	‐Oxygen vacancies: 2M_Zn_^+^ + 3V_O_•• ‐Zinc vacancies: 2M_Zn_^+^+ V_Zn_••	High lattice strain, reduced crystallite size	Strong inhibition of growth, increased specific surface area	Intermediate states in the bandgap, redshift toward visible, modulation of carrier density

a Substitution of Zn^2+^ by M^+^ (M^+^_Zn_) creates a charge deficit, which is compensated by the formation of oxygen vacancies V_O_••: M^+^_Zn_ + V_O_••→Zn_1−*x*_M^+^_*x*_O_1−*x*/2_.

b M^2+^ has the same charge as Zn^2+^ (M^2+^_Zn_), and the substitution is direct and forms a homogeneous solid solution: M^2+^_Zn_→Zn_1−*x*_M^2+^_*x*_O.

c Substitution of Zn^2+^ by M^3+^ creates an excess of positive charges (M^3+^_Zn_). The compensation occurs through zinc vacancies V^’’^_Zn_ and oxygen vacancies V_O_•• .

### 2.2 Effect of ZnO Doping on Electronic Structure

The objective is not simply descriptive but analytical, establishing a link between doping chemistry, induced defects, electronic structure, and photocatalytic activity [19]. The introduction of monovalent (M^+^), divalent (M^2+^), and trivalent (M^3+^) metal ions into the ZnO crystal lattice can occur via three distinct scenarios. The most common mechanism involves the partial substitution of Zn^2+^ ions by the dopant, accompanied by the creation of defects such as oxygen vacancies or other charge‐compensating defects to maintain electrical neutrality. At high dopant concentrations (excess), metal ions can segregate into secondary phases, thus limiting the solubility of the dopant in ZnO. Under moderate doping conditions, partial substitution generally occurs, depending on the synthesis method and the nature of the Zn^2+^ precursors. The predominant mechanism allows both dopant incorporation and controlled defect formation, thereby promoting photocatalytic activity.

Table 2 summarizes the effects of doping on the properties of ZnO. Undoped ZnO exhibits very low oxygen vacancy concentrations, a hexagonal lattice, a wide bandgap, rapid electron–hole recombination, limited radical production, and high structural stability. Monovalent dopants slightly increase vacancies and distort the lattice, resulting in a small reduction in the bandgap and moderate improvements in electron–hole separation and radical generation. Divalent dopants preserve the lattice structure with a slight decrease in the bandgap, allowing for a moderate improvement in photocatalytic activity while maintaining good stability. Trivalent dopants create large vacancies and significant lattice strain, reduce the bandgap, and greatly improve electron–hole separation and radical production but can slightly compromise structural stability depending on the doping level. Overall, doping modulates the electronic and photocatalytic properties of ZnO, optimizing activity while preserving lattice integrity.

**TABLE 2:** Effects of doping on the structural and electronic properties of ZnO highlighting how different types of dopants influence bandgap, electron–hole (e^−^/h^+^) separation, and radical production (^•^OH, ^•^O_2_^−^) [20, 21].

Formula	Oxygen vacancies V_O_••	*E*_g_, eV	e^−^/h^+^ separation	Production (^•^OH, ^•^O_2_^−^)
ZnO	Very low	3.20 (no intermediate states)	Rapid recombination	Low
Zn_1−*x*_M^+^_*x*_O_1−*x*/2_ (Li^+^, Na^+^, K^+^, Ag^+^)	Low (~0.5 per dopant)	Little change only for Ag^+^ (few localized states)	Slight improvement	Moderate
Zn_1−*x*_M^2+^_*x*_O (Co^2+^, Ni^2+^, Cd^2+^, …)	Very low	Little change (limited intermediate states)	Moderate	Moderate
Zn_1−*x*_M^3+^_*x*_O_1+*x*/2_ (Al^3+^, Fe^3+^, Cr^3+^, Bi^3+^, Ln^3+^)	High (~x/2 per dopant)	Decreases down to 2.6 (multiple intermediate states)/visible	Strongly improved	High

## 3 Synthesis‐Driven Doping Engineering and Defect Control

**FIGURE 2:**
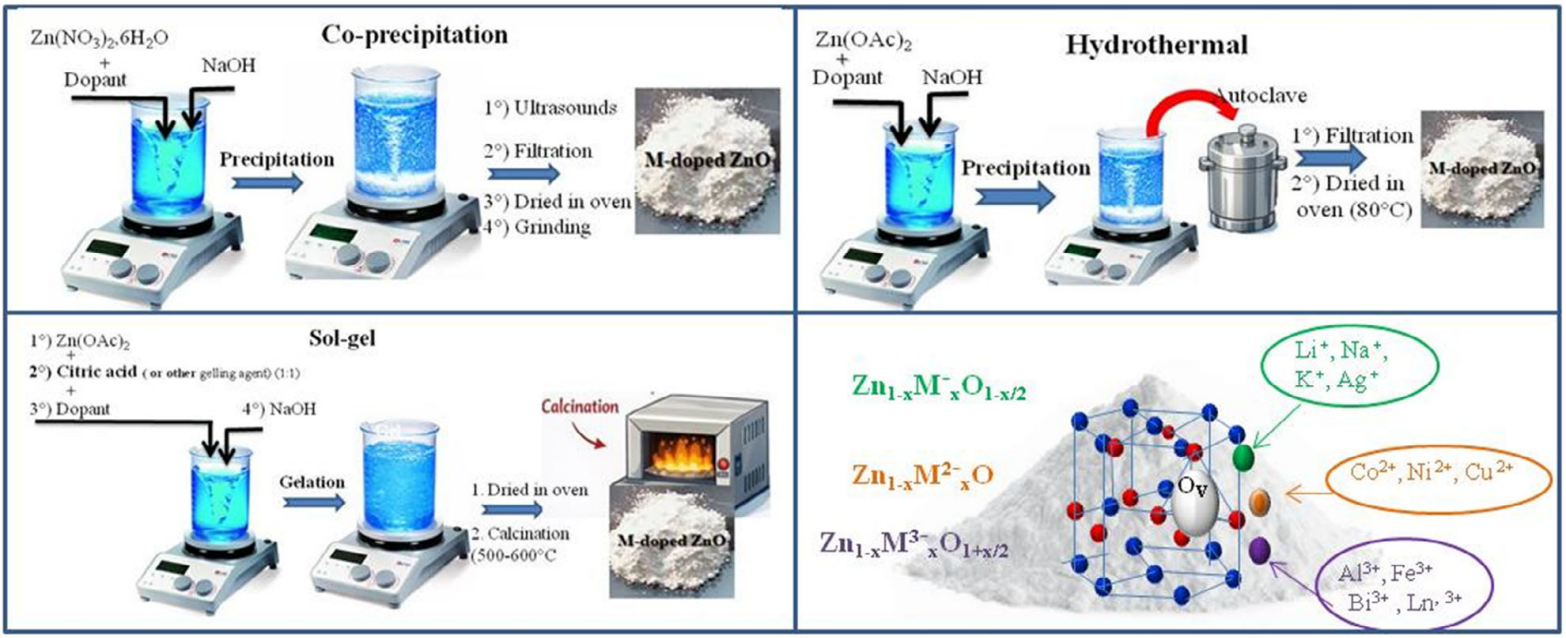
Experimental protocols for the synthesis of metal ion‐doped ZnO via coprecipitation, hydrothermal, and sol–gel methods.

The synthesis method strongly influences the incorporation of metallic dopants into the ZnO crystal lattice and the resulting defect distribution, particularly oxygen vacancies (V_O_), which are crucial for photocatalytic performance. Wet synthesis routes, including coprecipitation, hydrothermal methods, and sol–gel (Figure 2), are widely used to produce ZnO powders with controlled structural and surface properties. Coprecipitation is preferred for its simplicity, moderate cost, and the compositional control it allows [22]. The final material properties (morphology, crystallinity, specific surface area, and porosity) depend on the type and concentration of the precursor, the precipitating agent, the reaction temperature and duration, the pH, the addition rate, agitation, and postsynthesis treatments such as drying and calcination. These parameters determine particle size, lattice quality, and defect density, directly affecting photocatalytic activity [22, 23]. Hydrothermal synthesis, carried out under controlled temperature, pressure, and pH, allows for directed crystal growth and selective incorporation of dopants at energetically favorable sites, thus reducing undesirable structural defects. Reaction parameters regulate crystallinity, morphology, porosity, and surface defects—key factors for photocatalysis and adsorption [24, 25]. The sol–gel route promotes homogeneity at the molecular level between the dopant and the host network prior to calcination, thus facilitating the uniform substitution of Zn^2+^ and the controlled formation of oxygen vacancies [26, 27]. A moderate and well‐distributed V_O_ density promotes charge separation and the generation of ROS, while an excess or disorder of defects acts as recombination centers, limiting photocatalytic efficiency. Ultimately, optimizing doped ZnO requires a balance between crystallinity, dopant incorporation, and defect control to maximize charge separation and photocatalytic performance.

## 4 Experimental Verification of Oxygen Vacancy Formation via Metal Doping in the ZnO Lattice

In this review, we focus on trivalent metal ions, widely used to enhance the photocatalytic performance of materials. Their incorporation into the crystal lattice not only modifies the electronic structure of ZnO but also generates a high oxygen vacancy density, promoting charge separation and stimulating photocatalytic activity. Therefore, the study of trivalent doping is essential for understanding and optimizing the mechanisms underlying photocatalyst efficiency.

X‐ray diffraction (XRD), X‐ray photoelectron spectroscopy (XPS), and photoluminescence (PL) analyses show that the relative oxygen vacancy concentration increases with the valence and ionic radius of the dopant. XRD patterns reveal lattice distortions or peak shifts, indicating substitutional doping and defect formation, while XPS provides information on chemical state changes and relative oxygen vacancy concentrations, confirming that higher valence dopants with larger ionic radii favor defect generation. PL spectroscopy is commonly used to study the radiative recombination of electron/hole (e^−^/h^+^) pairs, as the PL intensity is directly proportional to the recombination rate of photoexcited carriers, resulting from better charge separation, often linked to the formation of defects or trap states in the bandgap [27, 28]. Increased V_O_ density correlates with stronger PL quenching, indicating more efficient charge separation and enhanced photocatalytic and optoelectronic performance [29].

### 4.1 Characterization of M^3+^‐Doped ZnO Powders Using XRD and EDS Mapping

**FIGURE 3:**
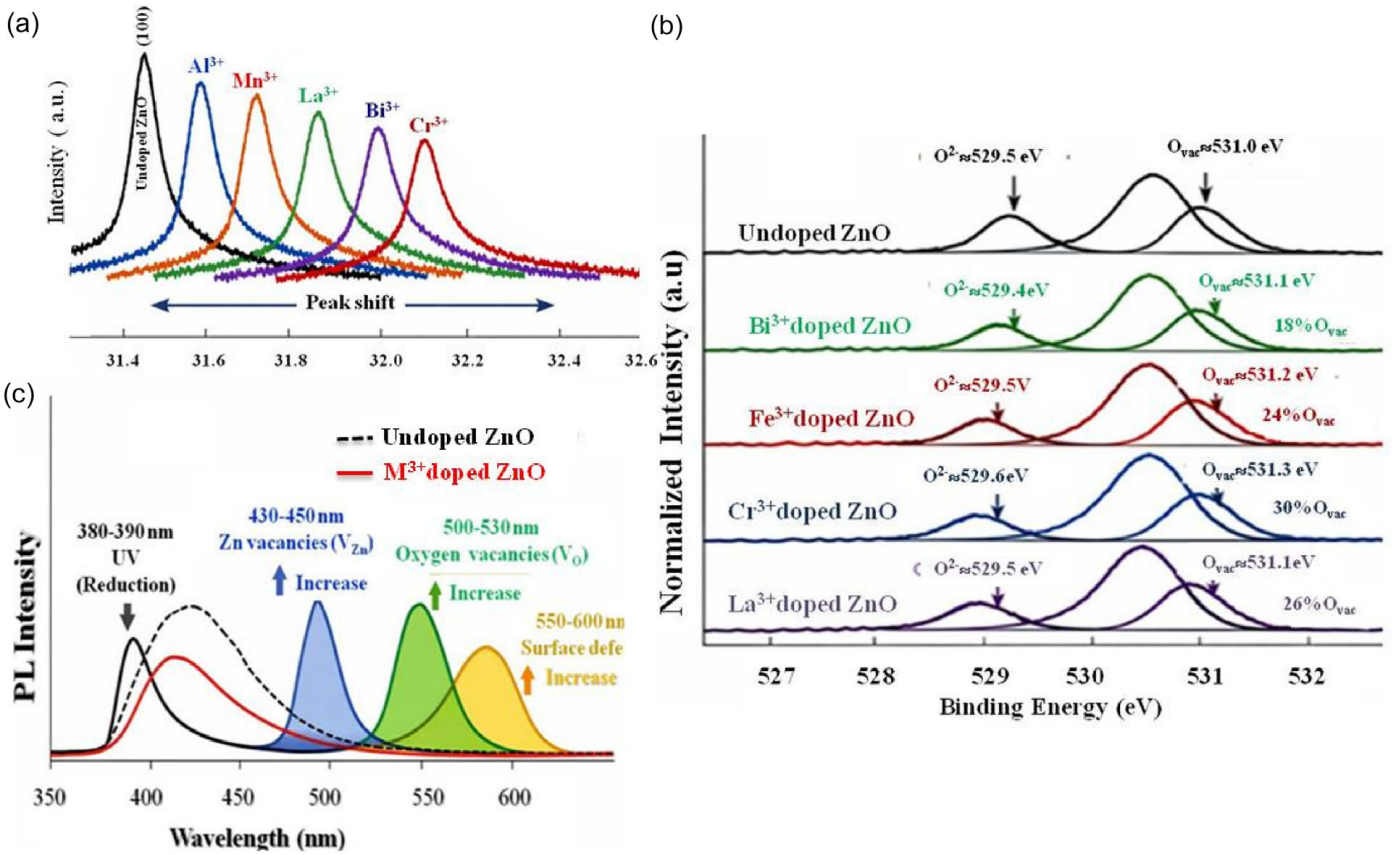
(a) XRD shift of (100) peak and illustrative spectra of (b) XPS O_1s_ and (c) PL of M^3+^ doped ZnO powders. Adapted with permission from Ref. [31, 32, 33] (2023 Elsevier, 2009 IOP, 2019 Elsevier).

Structural modifications induced by M^3+^ doping in ZnO have been widely investigated using XRD, which consistently confirms the preservation of the hexagonal wurtzite structure in most reported systems. The absence of secondary phases in many studies suggests that M^3+^ ions are generally incorporated into the ZnO lattice through substitutional mechanisms, although the solubility limit strongly depends on the nature and ionic radius of the dopant. A common feature reported in the literature is a systematic shift in diffraction peak positions and variations in peak broadening upon doping, which are typically attributed to lattice distortion, microstrain development, and crystallite size reduction. These structural changes are often associated with the formation of intrinsic defects, particularly oxygen vacancies, which are required to maintain charge neutrality in the case of aliovalent substitution. Doping ZnO with trivalent ions (M^3+^ = Al^3+^, Cr^3+^, Fe^3+^, La^3+^, Bi^3+^) partially substitutes Zn^2+^, creating a charge imbalance primarily compensated by oxygen vacancies (V_O_) [21, 30]. This leads to observable shifts in XRD peaks, which are closely related to both the ionic radius of the dopant and the resulting changes in the lattice parameters (Figure 3a). The ZnO crystal adopts a wurtzite structure characterized by the lattice parameters *a* and *c*. Substitution of Zn^2+^ (ionic radius ≈0.74 Å) with a smaller ion such as Al^3+^ (≈0.53 Å) generally induces a slight contraction of the lattice, resulting in a slight positive shift of the diffraction peaks (toward higher 2θ values). Conversely, bulkier dopants such as La^3+^ (≈1.03 Å) or Bi^3+^ (≈1.03 Å) expand the lattice, causing a negative shift (toward lower 2θ values) and a significant increase in *a* and *c* parameters. Intermediate‐sized transition metal ions, such as Fe^3+^ (≈0.65 Å) or Cr^3+^ (≈0.62 Å), produce moderate distortions, reflected by smaller Δ2θ shifts. These modifications to the crystal lattice are not purely geometric: The incorporation of trivalent dopants introduces a local charge imbalance, as Zn^2+^ is replaced by M^3+^. To maintain overall electroneutrality, the crystal compensates by forming oxygen vacancies (V_O_). Each trivalent ion substitution generates approximately one V_O_ vacancy for every two dopant ions to compensate for the excess positive charge. These oxygen vacancies occupy lattice sites, creating local distortions and further altering the lattice parameters. The interaction between the ionic size difference and charge compensation explains why the lattice parameters and diffraction peak positions are strongly influenced by the nature and concentration of the dopant.

In addition to XRD analysis, energy‐dispersive X‐ray spectroscopy (EDS) mapping has been extensively employed to enable the visualization of the spatial distribution of chemical species within particles or aggregates. Most reported studies indicate a homogeneous spatial distribution of M^3+^ ions within the ZnO matrix, confirming successful incorporation without significant phase segregation. However, it should be noted that EDS does not directly detect oxygen vacancies; their presence is generally inferred from structural distortions, compositional imbalance, or complementary spectroscopic techniques. Therefore, the combined XRD‐EDS mapping analysis not only confirms the successful incorporation of dopant ions into the ZnO matrix but also verifies their homogeneous distribution, which is essential for correlating the structural features with the functional properties of the material [31, 34, 35]. Based on EDS elemental mapping literature reports have confirmed that metal dopants (Fe, Cu, Ag, and Ce) are homogeneously distributed within ZnO nanostructures. This uniform incorporation enhances charge separation, modifies the band structure, and improves the generation of ROS (^•^OH, ^•^O_2_^−^), leading to superior photocatalytic activity.

### 4.2 XPS Analysis of Oxygen Vacancies in M^3+^‐Doped ZnO

XPS spectroscopy provides crucial information on the chemical composition, oxidation states, and defect structures of doped ZnO photocatalysts. It was used to study the effect of doping with trivalent metals (M^3+^ = Bi^3+^, Fe^3+^, Cr^3+^, La^3+^) on the surface oxygen states of ZnO [31, 32, 36, 37]. The spectra for all samples were deconvoluted into two main components: lattice oxygen (O^2−^) at approximately 529–530 eV and oxygen vacancies (V_O_) at 531–531.3 eV (Figure 3b). In pure ZnO, the contribution of oxygen vacancies (V_O_) is minimal, indicating a relatively stoichiometric surface area. After doping with M^3+^ ions, a notable increase in the relative intensity of the V_O_ peak was observed, reflecting the creation of additional oxygen vacancies. Among the doped samples, Cr^3+^ exhibited the highest proportion of oxygen vacancies, followed by La^3+^, Fe^3+^, and Bi^3+^, suggesting that the nature of the dopant and the ionic size significantly influence defect formation. These oxygen vacancies can serve as active sites for photocatalytic reactions, improving charge separation and promoting the generation of reactive species, which is crucial for enhancing the photocatalytic degradation of organic pollutants.

The XPS results thus provide clear evidence of the successful incorporation of M^3+^ ions into the ZnO lattice and their role in modulating surface defect concentrations, directly correlating with the observed photocatalytic performance. The analysis of the O_1s_ spectrum allows quantifying oxygen vacancies (V_O_), which play a central role in photocatalytic activity. These vacancies act as electron traps, inhibiting the recombination of photogenerated electron–hole pairs and thus promoting the generation of reactive species such as ^•^OH and ^•^O_2_^−^ radicals. A higher intensity of the V_O_ peak in XPS spectra is directly correlated with an increased concentration of these defects, often resulting in improved photocatalytic degradation rates of organic pollutants. Furthermore, XPS can reveal the successful incorporation of trivalent dopants into the ZnO lattice, which can modulate the electronic structure and promote charge separation [38]. Therefore, by combining XPS data with photocatalytic performance measurements, it is possible to establish a clear relationship: Materials with higher oxygen vacancy concentrations and optimized dopant incorporation tend to exhibit superior photocatalytic efficiency under UV and visible irradiation.

### 4.3 PL Analysis

PL spectroscopy is a powerful tool to probe the electronic structure and defect states of semiconductor materials such as ZnO [33, 39]. In doped ZnO, PL spectra typically exhibit near‐band edge (NBE) emission in the ultraviolet, corresponding to the recombination of free excitons, and broad visible emission associated with intrinsic defects, notably oxygen vacancies (V_O_) and interstitial atoms. The intensity and position of the visible emission peak directly indicate the concentration and nature of these defects. For example, ZnO doped with trivalent ions (Bi^3+^, Fe^3+^, Cr^3+^, La^3+^) displays enhanced yellow–green emission in the 500–550 nm range, generally attributed to the recombination of electrons from oxygen vacancies with holes in the valence band (Figure 3c) [40]. Consequently, PL spectroscopy not only confirms the formation of defect states but also provides complementary evidence for the role of these defects in photocatalytic activity, since oxygen vacancies can act as trapping sites for photogenerated electrons, reducing electron–hole recombination and enhancing the photocatalytic efficiency under UV or visible light [41].

Generally, strong visible PL emission, particularly in the green–yellow region, indicates a high density of oxygen vacancies or other defects, which act as electron traps. These traps can efficiently capture photogenerated electrons, thus reducing the recombination rate of electron–hole pairs. Lower recombination leads to a greater availability of reactive species, such as hydroxyl radicals and superoxide anions, which are responsible for the degradation of organic pollutants during photocatalytic reactions. Conversely, a very intense near‐band‐edge UV emission suggests fast electron–hole recombination, which can limit photocatalytic efficiency. Therefore, the PL spectra serve as an indirect but reliable indicator of photocatalytic activity: Doped ZnO samples with moderate visible emission, corresponding to optimal defect concentrations, often exhibit enhanced degradation rates of target pollutants under UV or visible irradiation, confirming the crucial role of defect engineering in tuning the electronic structure, charge carrier dynamics, and overall photocatalytic performance of ZnO‐based materials.

## 5 Bandgap Narrowing and Visible‐Light Activation

In its pure state, zinc oxide (ZnO) is a wide‐bandgap direct semiconductor with energy from 3.2 to 3.3 eV at room temperature, which limits its photon absorption to the ultraviolet range. The controlled introduction of trivalent ions (M^3+^) and oxygen vacancy‐type defects (V_O_) into the ZnO crystal lattice fundamentally alters its electronic structure [42]. When an M^3+^ ion substitutes for a Zn^2+^ ion in the lattice, intermediate energy levels are formed within the bandgap. These levels act as additional donor or acceptor states, thus reducing the minimum energy required to excite an electron from the valence band to the conduction band. This optical bandgap modification is often attributed to the combined effect of doping and the presence of defects such as oxygen vacancies, which increase the density of electronic states available within the gap. This reduction of the bandgap (*E*_g_) is advantageous for photocatalysis under solar light, as it enables direct photon absorption in the visible range (≈400–700 nm), thereby enhancing the generation of charge carriers (electrons and holes) through photon‐induced transitions. Various experimental and theoretical studies have reported optical bandgap (*E*_g_) values for ZnO doped with different trivalent metal ions (M^3+^), revealing a general trend toward bandgap reduction compared to pure ZnO (~2.6–3.2 eV) (Table 3). Al^3+^‐doped ZnO shows a slight decrease in *E*_g_ to around 3.0 eV under certain synthesis conditions, which is attributed to the formation of defects and extended energy states induced by doping. Cr^3+^‐doped ZnO exhibits a redshift in the absorption edge, with *E*_g_ values being reduced to approximately 3.0 eV or lower, enhancing visible‐light absorption. In the case of Fe^3+^ doping, moderate bandgap reductions have been observed (from 3.1 to ~2.8 eV) due to the presence of Fe^3+^ ions and associated defects, which improve optical absorption in the visible range. Although less widely studied, La^3+^ doping is known to induce lattice distortions and generate defects, effectively narrowing the bandgap and extending optical response into the visible spectrum. Finally, Bi^3+^‐doped ZnO shows a clear decrease in *E*_g_ with increasing Bi^3+^ concentration reaching up to 2.6 eV, introducing intraband electronic states that facilitate the absorption of lower‐energy photons, thereby enhancing visible‐light activity.

**TABLE 3:** Bandgap energy (*E*_g_) and oxygen vacancy (V_O_) percentage in pristine ZnO and trivalent‐ion‐doped ZnO.

Trivalent dopant (M^3+^)	Typical *E*_g_, eV^a^	Oxygen vacancy level (V_O_)^a^	Estimation method	Main origin of V_O_	Ref.
Undoped	3.20–3.30	5%–10%	XPS	Intrinsic defects	[43]
Al^3+^	3.1−3.20	8%–15%	XPS, PL	Limited charge compensation	[44]
Ga^3+^	3.15–3.3.25	10%–18 %	XPS	Slight lattice distortion	[45]
In^3+^	3.10 −3.20	15%–25%	XPS, PL	Charge imbalance + ionic size effect	[18]
Fe^3+^	2.80–3.10	25%–35%	XPS	3d states + charge compensation	[46]
Cr^3+^	2.60−3.00	30%–40%	XPS	d–d transitions, structural defects	[18]
Bi^3+^	2.60–3.00	35%–50%	XPS	Strong lattice distortion + Bi–O hybridization	[18]

a According to specified conditions.

The reported oxygen vacancy (V_O_) percentages are primarily estimated by XPS through deconvolution of the O_1s_ peak. This peak is generally decomposed into three components: lattice oxygen (O^2−^) with low binding energy, an intermediate component associated with oxygen vacancies or weakly bound oxygen species in oxygen‐poor media, and a high‐binding‐energy component linked to surface adsorbed species such as hydroxyl groups, water, or carbonates. The relative oxygen vacancy concentration is calculated from the ratio of the peak area corresponding to V_O_ to the total peak area of O_1s_ after background correction. These values should be considered semiquantitative, as they strongly depend on several experimental parameters. The nature of the trivalent dopant plays a key role: Dopants with small ionic radii and high oxygen affinity, such as Al^3+^ and Ga^3+^, mainly induce electronic charge compensation, thereby limiting oxygen vacancy formation. In contrast, transition metal or large‐sized dopants, including Fe^3+^, Cr^3+^, Mn^3+^, and Bi^3+^, promote structural defects and result in higher V_O_ densities. The dopant concentration significantly influences the compensation mechanism: Higher doping levels generally lead to increased oxygen vacancy formation and potential distortion of the crystal lattice. Furthermore, the synthesis route and heat treatment conditions have a decisive influence on the generation and stabilization of oxygen vacancies. High calcination temperatures and oxygen‐poor atmospheres promote V_O_ formation, while annealing in air or oxygen reduces their concentration. Therefore, oxygen vacancy percentages should be interpreted with caution, as comparative indicators rather than absolute values, especially when correlated with the optical and photocatalytic properties of materials.

## 6 Role of Trivalent Metal Ions and Oxygen Vacancies in ROS Generation

**FIGURE 4:**
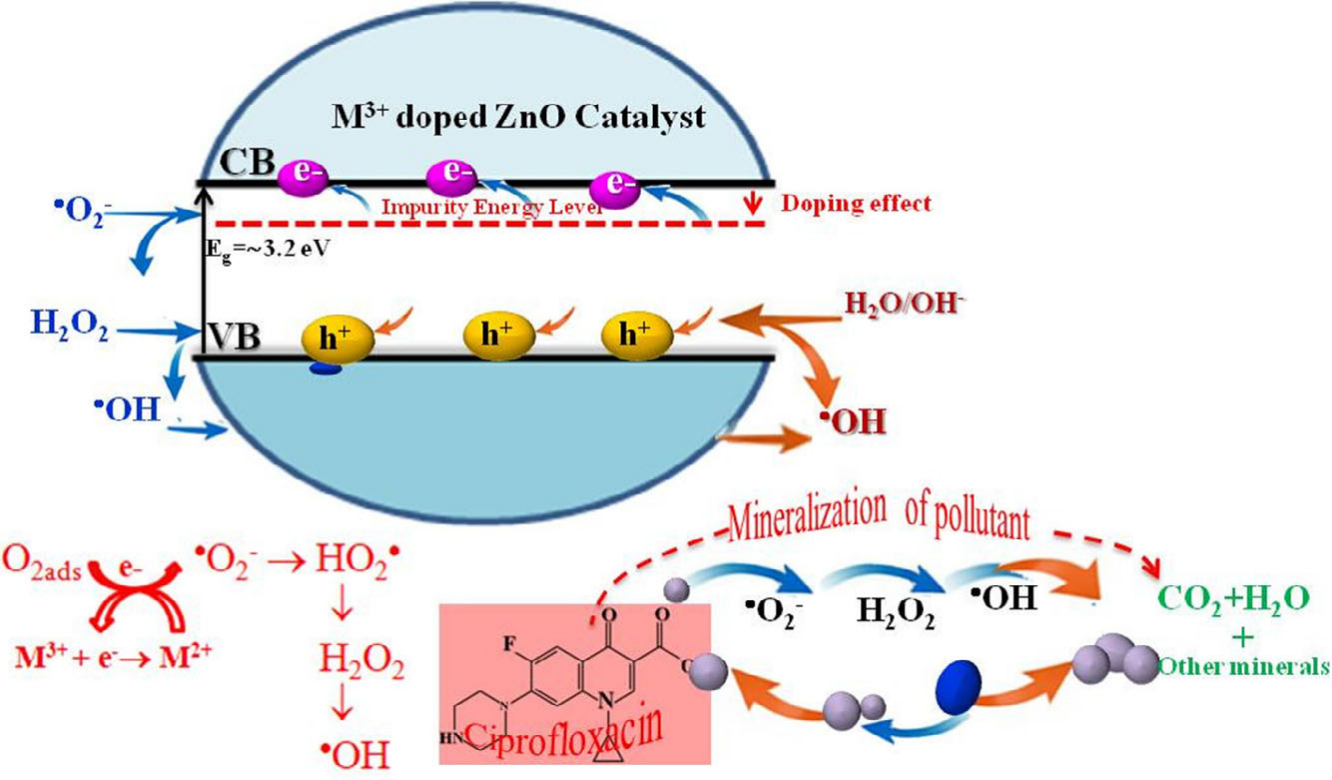
Mineralization process of antibiotic.

ZnO is a widely used semiconductor photocatalyst due to its wide bandgap about 3.2 eV, strong UV absorption, and efficient charge transport properties, enabling the photodegradation of various organic pollutants [47]. However, its practical application under solar irradiation is limited by low quantum efficiency, mainly due to the rapid recombination of photogenerated electron/hole (e^−^/h^+^) pairs and its weak absorption in the visible range [15, 48]. Holes (h^+^) oxidize water or hydroxide ions to produce highly reactive hydroxyl radicals (^•^OH), while electrons reduce oxygen molecules to form superoxide radicals (^•^O_2_^−^) and hydrogen peroxide (H_2_O_2_). These ROS are responsible for oxidizing and mineralizing organic pollutants into CO_2_, H_2_O, and other inorganic species [49]. However, this performance depends on conditions like pH, pollutant type, irradiation, and impurities, requiring process optimization [50, 51]. To overcome some limitations, metal ion doping has been extensively explored, particularly using monovalent [52], divalent [53, 54], and trivalent ions [15]. Among monovalent dopants, Ag^+^ largely enhances visible‐light absorption through the introduction of impurity levels near the conduction band and possible plasmonic effects, inducing a limited number of structural defects, resulting in a merely moderate suppression of charge carrier recombination. Divalent dopants partially replace Zn^2+^, slightly narrow the bandgap, and introduce shallow traps that enhance charge separation, yet they generate fewer defects and active sites than higher‐valence ions. However, trivalent ion doping (M^3+^: Al^3+^, Cr^3+^, Fe^3+^, Bi^3+^) has a pronounced effect on both the electronic structure and defect chemistry of ZnO. Substitution of Zn^2+^ by M^3+^ generates charge‐compensating oxygen vacancies (*V*_o_), which act as electron traps to reduce recombination, enhance charge carrier lifetime, and create intermediate energy states that enable visible‐light absorption. Additionally, these oxygen vacancies increase the number of surface active sites, facilitating the adsorption of reactants and promoting the generation of ROS. As a result, M^3+^‐doped ZnO exhibits superior photocatalytic efficiency compared to mono‐ or divalent‐doped ZnO, particularly under solar or visible‐light irradiation. The synergistic effect of trivalent doping involves the following: (i) bandgap engineering, enabling absorption of lower‐energy photons; (ii) enhanced charge separation, via electron trapping at oxygen vacancies; (iii) increased surface activity, due to defect‐induced adsorption sites; and (iv) improved ROS generation, especially •OH and •O_2_^−^, driving rapid and selective pollutant degradation (Figure 4).

Doping introduces foreign atoms into ZnO's lattice, altering its electronic, optical, and structural properties by creating lattice strain, charge imbalances, or new energy states that enhance material performance. Transition metal dopants (Mn, Fe, Co, Ni, and Cu) create localized states in ZnO, reducing its bandgap and enabling visible‐light‐driven photocatalytic activity [14]. Moreover, it was shown that Fe incorporation suppresses charge recombination in the ZnO composite by effectively capturing conduction band electrons [55]. Visible‐light‐active Cu and Ag‐doped ZnO show enhanced photocatalytic performance. In Cu‐doped ZnO [56, 57], Cu‐induced oxygen vacancies improve light absorption, reduce charge recombination, and increase active sites Ag doping enhances surface area, porosity, and morphology, while band structure modification enables visible‐light absorption, crucial for efficient solar‐driven photocatalysis [58].

Table 4 outlines the different steps of the photocatalytic mechanism of M^3+^‐doped ZnO in the presence of oxygen vacancies (*V*_o_). The process begins with photoinduced excitation, where photon absorption by M^3+^:ZnO generates electrons and holes (hν → e^−^+h^+^), facilitated by bandgap narrowing and visible‐light activation. The free electrons can then be trapped by the dopant metal ions (M^3+^+e^−^→ M^2+^), suppressing e^−^/h^+^ recombination and enhancing charge separation. Oxygen vacancies play a critical role in stabilizing the excited charges (V_O_ + e^−^ → V_O_^−^) and promoting selective adsorption of molecular oxygen (O_2_(g) → O_2_(ads)/V_O_). This adsorption enables the dominant formation of superoxide radicals (e^−^ + O_2_ → ^•^O_2_^−^), which are key ROS in the oxidation process. The metal redox cycle (M^2+^ + O_2_ → M^3+^ +^•^O_2_^−^) further amplifies electron transfer, sustaining continuous radical generation. Simultaneously, the photogenerated holes drive the direct oxidation of water or hydroxide ions (h^+^+H_2_O/OH^−^→^•^OH), producing highly oxidizing hydroxyl radicals. Secondly, the generation of hydrogen peroxide (2^•^O_2_^−^ + 2H^+^ → H_2_O_2_ + O_2_) and its subsequent activation by the dopant metal (M^2+^+H_2_O_2_→M^3+^+OH + OH^‐^) contribute to the enhanced production of hydroxyl radicals (•OH). Finally, adsorbed pollutants are degraded by these ROS (Pollutant + ROS → CO_2_ + H_2_O), resulting in complete mineralization. This mechanism highlights the synergistic effect of metal doping and oxygen vacancies on ROS generation and stabilization, thereby optimizing the photocatalytic efficiency of ZnO under visible light.

**TABLE 4:** Mechanistic overview of the role of M^3+^ ions and oxygen vacancies in ZnO photocatalysis [54, 55, 56, 57, 58].

Step	Physicochemical process	Associated equation	Key role of M^3+^/V_O_
1	Photoinduced excitation	ZnO:M^3+^ + hν→e^−^+h^+^	Bandgap narrowing and visible‐light activation
2	Electron trapping	M^3+^+e^−^→ M^2+^	Suppression of e^−^/h^+^ recombination
3	Vacancy‐assisted trapping	V_O_+e^−^→V_O_^−^	Charge stabilization
4	Oxygen adsorption	O_2_(g) → O_2_(ads)/V_O_	V_O_ promotes selective oxygen adsorption
5	Superoxide formation	e^−^ + O_2_ →•O_2_^−^	Dominant ROS generation
6	Metal redox cycle	M^2+^ + O_2_ → M^3+^ + •O_2_^‐^	Amplification of electron transfer
7	Hole‐driven oxidation	h^+^ + H_2_O / OH^−^ → •OH	Generation of strong oxidizing ROS
8	H_2_O_2_ formation	2•O_2_^−^ + 2H^+^ → H_2_O_2_ + O_2_	Secondary ROS formation
9	H_2_O_2_ activation	M^2+^ + H_2_O_2_ → M^3+^ + •OH + OH^−^	Additional •OH production
10	Pollutant oxidation	Pollutant + ROS → CO_2_ + H_2_O + ions	Final mineralization

## 7 Photocatalytic Performance of Metal‐Doped ZnO: Mechanistic Insights and Pollutant Degradation

ZnO is a widely studied photocatalyst for water treatment due to its low cost, nontoxicity, chemical stability, and strong oxidizing power. However, its wide bandgap limits activation to the UV region, and rapid e^−^/h^+^ recombination reduces quantum efficiency. Metal doping is a common strategy to overcome these limitations, as it can (i) modify the electronic structure by introducing intraband energy levels, (ii) create structural defects such as oxygen vacancies, (iii) enhance charge separation through selective electron or hole trapping, and (iv) extend optical absorption into the visible range. These effects collectively enhance the generation of ROS (^•^OH, ^•^O_2_^−^, HO_2_^•^), responsible for pollutant mineralization.

### 7.1 Metal‐Doped ZnO

Monovalent ions (Ag^+^, Li^+^, Na^+^, and K^+^) induce minor lattice distortions where Ag^+^ is particularly effective, forming either lattice substitutions or metallic Ag^0^ nanoparticles that produce localized surface plasmon resonance (LSPR), improving visible‐light absorption and electron transfer [59]. Photocatalytic studies report > 90% degradation of dyes like methylene blue (MB), rhodamine B (RhB), and methyl orange (MO) under UV–Vis light. Li^+^ and Na^+^ doping slightly increase defect concentration, yielding modest improvements under UV light; however, oxygen vacancy generation remains limited, and e^−^/h^+^ recombination remains a key limitation. Most divalent ions have ionic radii close to that of Zn^2+^, facilitating their substitution into the crystal lattice. Cu^2+^ introduces acceptor levels near the valence band, enhancing visible‐light absorption and degrading azo dyes, antibiotics, and phenolics. Ni^2+^ and Co^2+^ improve charge separation but may act as recombination centers at high concentrations. Mn^2+^ and Fe^2+^ promote oxygen vacancy formation and participate in redox cycles (Mn^2+^/Mn^3+^, Fe^2+^/Fe^3+^), boosting ROS generation [60].

Table 5 summarizes the photocatalytic performance of metal‐doped ZnO against selected pollutants. Two percent of Mn–ZnO degraded 66% of methyl green under sunlight in 60 min and 99% of MB under UV in 120 min; 5% Cu–ZnO achieved 75% degradation of methyl green under visible light in 50 min; 6% Mg–ZnO reached 99.38% degradation of basic fuchsin under UV in 100 min. Trivalent ions (Al^3+^, Ga^3+^, Cr^3+^, Fe^3+^, Bi^3+^, La^3+^, and Ce^3+^) create charge imbalance in the lattice, promoting oxygen vacancy formation and introducing defect states within the bandgap, which enhances ROS generation and visible‐light absorption. Fe^3+^‐doped ZnO efficiently degraded MO (94% in 180 min) and 2‐chlorophenol (~85% in 2 h). Cr^3+^‐doped ZnO achieved 98% MO degradation in 100 min under UV–Vis. Al^3+^ doping reduced the bandgap (~2.8 eV), enabling 91% MO degradation in 40 min. Bi^3+^ and La^3+^ improved surface adsorption and electron–hole separation, yielding 46%–98% degradation for dyes such as MO, Congo Red, and MB. Ce^3+^ doping under visible light reached 82%–98.4% degradation for DB71, MB, and tetracycline, facilitated by Ce^3+^/Ce^4+^ redox cycles. Codoped systems (e.g., 3%La‐5%Cu‐doped ZnO and %Bi‐1%Ce‐doped ZnO), exhibited synergistic effects, achieving 96%–98% degradation of the pollutants under study.

**TABLE 5:** Photocatalytic performance of metal‐doped ZnO versus selected pollutants.

Metal‐doped ZnO	*E*_g_, eV	Pollutant^a^	Source	Photocatalytic condition	Degradation/time	Ref.
4%Ag^+^–ZnO	2.79	MO	UV (450 W)	C_0_ = 10 ppm	86.5% in 120 min	[61]
2%Mn^2+^–ZnO	—	MG	Sunlight	C_0_ = 20 ppm, dose = 0.1 g/L	66.4% in 60 min	[62]
2%Mn^2+^–ZnO	—	MB	UV	0.3 g/L —	99% in 120 min	[63]
5%Cu^2+^–ZnO	—	MG	Vis		75% in 50 min	[64]
6%Mg^2+^–ZnO	3.16	BF	UV	C_0_ = 10 ppm, pH 6, dose 0.1 g	99.3% in 100 min	[65]
10% Co^2+^–ZnO	2.75	CIP	Vis‐LED	30 ppm, pH = 7, dose 2 g/L	94% in 90 min	[66]
6%Ni^2+^–ZnO	—	MG	UV	C_0_ = 15 ppm	76% in 60 min	[67]
2%Fe^3+^.ZnO	3.1	MO	UV (150 W)	pH ~ 8, C_0_ = 10 ppm, dose 1 g/L	94% in 180 min	[68]
1%Fe^**3+**^–ZnO	2.87	CPh	Solar (23 Wm^−2^)	C_0_ = 50 ppm, dose = 1 g/L	∼85% in 2 h	[69]
10% Co^3+^–ZnO	2.96	CIP	Vis‐LED 150 W	C_0_ = 20 ppm, pH 7, dose = 1 g/L	99% in 60 min	[70]
1%Cr^3+^ **–**ZnO	2.28	MO	UV	C_0_ = 10 mg L^−1^	98 % in 100 min	[71]
10% Al^3+^–ZnO	2.8	MO	UV_10 W	C_0_ = 50 ppm	91% in 40 min	[72]
5%Bi^3+^–ZnO	—	MO	UV‐300 W	C_0_ = 10 ppm, dose = 1 g/L, pH 7	46.2 % in 1 h	[60]
5%Bi^3+^–ZnO	3.0	CR	UV	Dose = 1.6 g/L, C_0_ = 0.1 mM	∼80% in 1 h	[73]
7%Bi^3+^–1%Ce‐ZnO	2.83	MB	UV	2 mL of H_2_O_2_, *m* = 0.01 g,	98% in 2 h	[74]
5%Ce^3+^–ZnO	2.89	DB71	Vis	C_0_ = 50 ppm, dose = 0.1 g, pH 5	98.4%	[75]
7%La^3+^–ZnO	3.17	MB	UV (15 W)	C_0_ = 1.510^−5^ M, pH 6.5	93% in 2 h	[76]
5%La^3+^–ZnO	2.98	MB	Solar	C_0_ = 20 ppm	98% in 90 min	[12]
3%La^3+^–5%Cu‐ZnO	3.28	CIP	UV‐125 W	Dose = 0.5 g/L, C_0_ = 10 ppm	96% in 150 min	[77]
La^3+^–ZnO 2%Eu^3+^–ZnO	—	RB5	UV‐BLB18 W	C_0_ = 20 ppm, *m* = 0.125 g, pH ∼ 6	92% in 30 min 85% in 30 min	[78]

a Methyl green (MG), methylene orange (MO), methylene blue (MB), basic fuchsin (BF), rhodamine B (RhB), direct blue 71 (DB71), Congo Red (CR), reactive black 5 (RB5), 2‐chlorophenol (CPh), ciprofloxacin (CIP), dose: catalyst dose, C_0_: initial conc. of pollutant.

### 7.2 Coupling of Semiconductors With ZnO

The photocatalytic performance of the MgO/ZnO hybrid material is significantly enhanced through the synergistic coupling of some semiconductors [12]. The system exhibits an extended light absorption into the visible region (≥400 nm) and a reduced effective bandgap compared to the individual parent semiconductors, thereby increasing photon utilization. Charge carrier dynamics are optimized within the heterojunction: photoinduced holes are confined in the valence band of the wide‐bandgap ZnO, while electrons are trapped in the conduction band of the narrow‐bandgap MgO, resulting in prolonged charge carrier lifetimes. This spatial separation effectively suppresses the recombination of photoexcited charge carriers, a primary factor limiting photocatalytic efficiency. The combination of these effects leads to accelerated and more efficient degradation of dye molecules under visible‐light irradiation, highlighting the practical potential of ZnO‐based photocatalysts in advanced environmental applications [79]. Chang et al. also fabricated BiOCl/ZnO p–n heterojunctions with exposed (101) facets, showing superior visible‐light photocatalysis due to enhanced absorption, larger surface area, and built‐in junction potential [80].

Carbon‐based materials including graphite, carbon fibers, carbon nanotubes, graphene, carbon black, activated carbon, and fullerenes are widely used as catalyst supports due to their high surface area, tunable porosity, thermal conductivity, neutral surface, and rich surface functionalities. Among second‐generation photocatalysts, coupling ZnO with graphitic carbon nitride (g‐C_3_N_4_) has proven highly effective in enhancing visible‐light (Vis) absorption and photocatalytic performance. g‐C_3_N_4_ is a two‐dimensional material with a bandgap of 2.65 eV, chemical stability, tunable electronic structure, low cost, and facile synthesis. Incorporating ZnO reduces the composite's bandgap and promotes efficient dye degradation via a Z‐scheme mechanism. For instance, Kalisamy et al. reported S‐doped ZnO/g‐C_3_N_4_ achieving 93% removal of methylene blue and rhodamine B within 80 min, maintaining stability over five cycles, thanks to accelerated hydroxyl radical generation from enhanced electron–hole separation [81]. Bandgap measurements confirmed the heterojunction effect (g‐C_3_N_4_: 2.66 eV; ZnO: 3.2 eV; ZnO/g‐C_3_N_4_: 2.63 eV). Similarly, Shemeena and Binitha achieved complete degradation of 10 mg/L Congo red in 50 min using 25 mg of ZnO/g‐C_3_N_4_ [82], while Wu et al. reported a ternary ZnO/Fe_3_O_4_/g‐C_3_N_4_ system attaining 98% degradation of 30 mg/L alizarin yellow R in 150 min [83]. These studies highlight the significant potential of ZnO/ g‐C_3_N_4_ composites and multicomponent heterojunctions as efficient visible‐light photocatalysts for water treatment applications [84]. Zhang et al. studied g‐C_3_N_4_/ZnO/graphene aerogel and g‐C_3_N_4_/ZnO composites for rhodamine B photodegradation [85]. The materials exhibited high efficiency (81%–92%) under UV and visible light, due to hierarchical structures, synergistic effects, and the main action of superoxide and hydroxyl radicals [80]. ZnO heterojunctions with CuO [86], MgO [81], BiOCl [80], AgIO_4_ [87], ZnFe_2_O_4_ [88], and BiVO_4_ [87] enhance photocatalysis under visible light by improving visible‐light absorption, reducing e^−^/h^+^ recombination, and generating active sites for degrading dyes such as rhodamine B or methylene blue. Bandgap reduction, Ag plasmonics, and small particle size contribute to rapid degradation and good reusability for water treatment. Zhu et al. prepared Fe–Cu–ZnO/graphene oxide composites exploiting both adsorption and photodegradation for dye removal, achieving 99% degradation of 50 mg/L under visible light due to metal doping and the high specific surface area of graphene oxide [89]. Oppong et al. synthesized a La–ZnO/GO composite, whose enhanced photocatalytic performance was attributed to GO acting as an electron scavenger, providing stability and reusability (98% performance versus 18% for pure ZnO) [90]. Zarrabi et al. confirmed that GO facilitates electron transfer, preventing charge recombination and achieving 98%–100% degradation of dyes within 180 min [91]. Other studies include ZnO–Ag composites in an amino‐silicate sol–gel matrix, Ce^3+^‐doped ZnO@chitosan, and Cd^2+^/Ag^+^‐doped ZnO nanomaterials with PVP or PPy, which improved charge separation and reduced bandgap energy [92, 93].

The future of ZnO‐based photocatalysis requires greener synthesis methods. Although many studies report ZnO nanoparticles with varied morphologies, improvements in crystallinity and sphericity are still needed to enhance photocatalytic performance. Research should prioritize real pollutants, such as pesticides, pharmaceuticals, and endocrine disruptors, rather than easily degradable model dyes. A deeper understanding of mineralization pathways at the photocatalyst‐pollutant interface is essential. Operational challenges, including poor catalyst recovery and decreased activity of recycled or immobilized photocatalysts, must be addressed. Finally, theoretical tools and meta‐analyses can guide the design of new catalysts, reducing trial‐and‐error experimentation and promoting more sustainable photocatalysis research. Nanostructured ZnO is a promising alternative to TiO_2_ for visible‐light‐driven photocatalytic water treatment. Its efficiency critically depends on synthesis methods, particle size, and morphology, with sol–gel routes offering precise control at low cost. Enhancing performance focuses on suppressing charge recombination through metal/nonmetal doping and coupling with other semiconductors or carbon‐based materials, rather than solely reducing the bandgap. Careful optimization of dopant types and concentrations maximizes photocatalytic activity. Overcoming mass‐transfer limitations remains key for scaling up ZnO photocatalysts from laboratory studies to industrial and municipal water treatment applications.

## 8 Economic Considerations of Metal‐Doped ZnO and ZnO‐Based Composites

The cost of metal‐doped ZnO materials and their composites with various semiconductors is a key factor for their development in photocatalysis. The price of pure ZnO depends on particle size and purity, typically ranging from $50 to $300 per 100 g for small laboratory quantities and from $18 to $180 per kg for commercial‐scale amounts. The incorporation of low percentages (max 10%) of common metal dopants adds a negligible cost, whereas noble metals can significantly increase the price. The formation of ZnO/semiconductor composites with TiO_2_, CdS, SnO_2_, ZnS, graphene oxide (GO), or g‐C_3_N_4_ also affects the final cost, which can be approximated as a weighted average of the components. For example, a Cu‐doped ZnO composite with GO remains affordable (~$70–$80/kg), while the inclusion of specialized semiconductors such as g‐C_3_N_4_ incurs a slight additional cost. These estimates indicate that, despite doping or composite formation, ZnO materials remain relatively accessible, supporting their widespread adoption in photocatalytic and environmental applications.

## 9 Conclusion

Recent studies on doped ZnO demonstrate that the nature and valence of the dopant ions play a crucial role in enhancing photocatalytic performance. In particular, trivalent ions (M^3+^) promote the formation of a significant number of oxygen vacancies, modifying the electronic structure of ZnO and facilitating the separation of electron–hole pairs. This reduction in recombination increases the generation of hydroxyl and superoxide radicals, which are essential for pollutant oxidation, while extending light absorption into the visible range and enabling catalyst activity under solar irradiation. These structural and electronic modifications directly translate into faster and more efficient photodegradation of dyes and pharmaceutical micropollutants, with numerous studies reporting near‐complete mineralization of contaminants. Precise control of the doping also allows tuning of surface properties and ZnO reactivity, optimizing performance for different types of pollutants. In this context, trivalent ion doping emerges as a key strategy for developing high‐performance, stable, and adaptable ZnO photocatalysts. The combination of electronic modification, increased oxygen vacancies, and improved light absorption constitutes a major lever for achieving complete degradation of organic pollutants. These results highlight the direct relationship between dopant valence, structural defect generation, and photocatalytic efficiency. Finally, this in‐depth understanding of doping mechanisms paves the way for the design of optimized ZnO photocatalysts capable of efficiently treating contaminated water on a large scale, contributing sustainably to environmental remediation and offering real potential for industrial and ecological applications.

## Funding

This study was supported by the Natural Sciences and Engineering Research Council of Canada (RGPIN‐2020‐07016).

## Conflicts of Interest

The authors declare no conflicts of interest.

## Data Availability

Data sharing is not applicable to this article as no datasets were generated or analyzed during the current study.
